# Diabetic levels of glucose increase cellular reducing equivalents but reduce survival in three models of 661W photoreceptor-like cell injury

**DOI:** 10.1186/s12886-015-0164-2

**Published:** 2015-12-09

**Authors:** Christopher J. Layton

**Affiliations:** Ophthalmology Research Unit, Gallipoli Medical Research Institute, Newdegate St, Greenslopes, Brisbane, 4120 Australia; School of Medicine, University of Queensland, Herston Rd, Herston, Brisbane, 4006 Australia; Ophthalmology Department, Greenslopes Hospital, Newdegate St, Greenslopes, Brisbane, Australia

**Keywords:** Photoreceptor, Neuroprotection, Glucose, Diabetic retinopathy, Diabetes

## Abstract

**Background:**

The effect of excess glucose on retinal cellular health remains controversial, and cellular reducing equivalents, as indicators of cellular energy production, are widely used as substitute indicators of retinal cellular health. These investigations hypothesised that excess energy substrate availability, as occurs in the diabetic retina, increases the susceptibility of retinal neurons to injury in the presence of increased cellular reducing equivalents.

**Methods:**

The response of 661W cells to phototoxicity, oxidative stress induced by H_2_O_2_ and apoptosis induction by staurosporine was characterised in the presence of 5mM glucose and B27 defined media without insulin. Cellular insult was produced by phototoxicity, H_2_O_2_ and the apoptosis induction agent staurosporine. The effect of physiologically relevant alterations in environmental glucose on cellular reducing equivalents was assessed by MTT dye reduction and NAD(P)H assays, and cell survival was assessed via caspase 3/7 activation and Annexin V/PI flow cytometry.

**Results:**

661W photoreceptor-like cells underwent dose dependent cell death primarily by apoptosis in response to phototoxic insult, H_2_O_2_, and staurosporine by all measures of cellular viability. Exposure of cells to 25mM glucose (diabetic-type conditions) increased cell death in response to all insults as measured by caspase 3/7 activation and Annexin V/PI flow cytometry. Cellular reducing equivalents were nonetheless increased in all models of injury in the presence of excess glucose. The mechanism of this increase was partly due to increased NADPH but not NADH levels in the presence of 25mM glucose.

**Conclusions:**

Acute exposure to 25mM glucose decreased the resilience of 661W photoreceptor-like cells to a range of cellular stressors whilst maintaining or increasing cellular reducing equivalents, partly be increasing NADPH levels. This shows that in 661W cells, diabetic levels of glucose decrease cellular resilience to injury. The decoupling of cellular reducing equivalents levels from cell survival has important implications when investigating the mechanisms of neuronal damage in diabetic retinal neuropathy.

## Background

Diabetic retinopathy is becoming increasingly recognised as a panretinal disease beginning early in diabetes with alterations in neuronal function (“diabetic retinal neuropathy”) prior to the onset of a diabetic retinal vasculopathy [1, 2]. This vasculopathy is eventually observable ophthalmoscopically and is the cause of clinical vision loss [3]. Since diabetic retinal neuropathy occurs in the presence of grossly normal retinal perfusion [3], understanding the mechanisms causing diabetic retinal neuropathy are important in attempts to arrest the ocular manifestations of diabetes prior to the onset of the vision threatening vasculopathy.

As the changes associated with diabetic retinal neuropathy manifest early in diabetes, an understanding of the mechanisms of retinal neuronal damage in the disease demands that the effect of high environmental glucose on the resilience of retinal neuronal cells is explored. Glucose is a fundamental source of energy for retinal cells [4], and it seems intuitive that the presence of abundant glucose will assist retinal neuronal survival and function, however many studies have suggested that excess mitochondrial free radical production in response to higher flux through the electron transport chain in conditions of excess energy substrate is the cause of diabetic complications [5, 6]. Whether glucose promotes survival or cell death in retinal cells remain controversial: a protective effect of glucose on stressed retinal cells has been confirmed in conditions of ischaemia [7], respiratory chain inhibition [8] and glaucoma [9], however studies in primary retinal culture show glucose inhibits the protective effect of neurotrophic factors [10], and other CNS neurons have been shown to be less resilient to insult in the presence of high environmental glucose [11].

An increasingly common method of investigating the mechanisms of diabetic complications in the retina is through exploring cellular survival in in vitro retinal cell models [10, 12, 13]. Methods commonly used to assess the health of these in vitro models of retinal cells include neurite outgrowth assays, cell viability dyes [14], proliferation assays [15], apoptotic marker detection such as activated caspase expression [16], phosphatidylserine expression (annexin V binding) [17], and detection of DNA breakdown (TUNEL protocols) [18]. Most of these methods have been validated in culture systems containing 25mM glucose. Unfortunately, it is likely that not all of these methods are valid surrogate markers of cell health in investigations which alter the concentrations of energy substrates. Proliferation assays in particular, are in widespread use and utilise cellular reducing equivalents as markers of metabolic activity and an indicator of total cell viability. Cellular reducing equivalents have been shown to be closely coupled to cellular survival and cell number in multiple separate investigations [19–21]. They provide a reflection of cellular redox activity which is principally a product of cellular energy metabolism, which also provides the primary source of reactive oxygen species which are thought to be important in the pathogenesis of diabetic complications [22]. It is therefore possible that altering the availability of the energy substrate glucose will alter the correlation between cellular reducing equivalents and cell survival, and any uncoupling is therefore likely to be an unexpected confounding factor in experiments involving cellular health in diabetes.

Photoreceptors have been shown to be one of the primary cell types affected in diabetic retinal neuropathy, with increased levels of photoreceptor apoptosis seen in histological sections of animal models of diabetes [23] and thinning of the photoreceptor layer noted on OCT scanning in human subjects with diabetes [24]. In this study, 661W cells were used to investigate the effect of physiologically relevant raised levels of glucose on the resilience of photoreceptor-like cells to a wide range of cellular stressors to test the hypothesis that retinal cellular reducing equivalents are increased by high glucose substrate levels and that their level in such conditions is independent of cellular survival after retinal cell injury.

## Methods

All work was performed at the Gallipoli Medical Research Institute and was covered by an exempt dealing agreement with the University of Queensland Ethics and Biosafety Committees. Unless otherwise noted, materials were obtained from Sigma Aldrich (Sydney, Australia).

### 661W Cell culture

Passage 27 661W retinoblast cell cultures were seeded at 1 x 10^6^ cells per mL and grown at 37 °C and 5 % CO_2_ in DMEM supplemented with 5mM glucose, 1mM sodium pyruvate, GlutaMAX and 10 % foetal bovine serum (FBS), (Life Technologies, Melbourne, Australia) and characterised as mouse photoreceptor-like cells expressing opsin MW/LW. Experimental cultures were washed in phosphate buffered saline once 50 % confluent and allowed to just reach just reach confluence in the same media supplemented by defined media (B27 without insulin, Life Technologies, Melbourne) in the place of FBS. Cells maintained their morphology, growth rates and photoreceptor-like differentiation when supplemented by B27 media, and could be successfully passaged in this environment without serum supplementation. To help further standardise cell numbers in each well all experiments were undertaken just as 100 % confluence was achieved, and all experiments were performed on cells within 5 passages of characterisation. Additional glucose was added at the beginning of experimental treatments to minimise exposure time to the injuries of phototoxicity, oxidative stress (H_2_O_2_) and apoptosis (staurosporine), which were modified from the protocols of Kanan [25], Kunchithapautham [26], and Daudt [27] respectively.

### Phototoxicity

Phototoxic damage to 661W cell cultures was induced by treatment with glucose and 9-cis retinal immediately before being exposed to white light at 20,000 lux from below provided by a NorthStar 10,000 broad spectrum light box (Alaskan Northern Lights, Reno, NV). Ambient temperature of the cultures was 37 °C during treatment and cells were exposed to atmospheric gases. Following treatment, cells were removed from the light source and cell viability assays performed without illumination at 37 °C and 5 % CO_2._

### Cellular reducing capacity

Cellular reducing equivalents in conditions of cellular stress was assessed by the (3,4,5 – dimethylthiazol-2-yl)-2,5-diphenyltetrazolium bromide (MTT) reduction assay modified from that of Mossman [15] and as described previously [10]. Briefly, cells were subjected to the appropriate treatments and then MTT was added to the wells at a final concentration of 0.5 mg/mL for one hour at 37 °C. The medium was subsequently carefully removed so as not to disturb remaining cells, and the reduced MTT (blue formazan product) was solubilised by adding 100 μL of dimethyl sulphoxide to each well. After agitation of the plate for 15 min, the optical density of the solubilised formazan product in each uncovered well was measured using an automatic microplate reader (Tecan F200, Mannedorf, Switzerland) with a 540nm wavelength and a top reading protocol.

### Quantification of reduced NADP(H) and NAD(H)

Cells were grown in black walled 384 well plates and treated according to the protocol of each injury model. NADPH, NADH and their associated oxidised forms were quantified by fluorometric assay (Abcam, Cambridge, MA). Briefly, cells were lysed in 20 μL cell lysis buffer and treated with 5 μL NADPH/NADH/NADP+/NAD+ extraction solution for 15 min at room temperature before being neutralised by an equal volume of complementary extraction solutions. 15 μL NADPH and NADH reaction solution was added and uncovered well fluorescence was measured with an automatic microplate reader (Tecan F200) with a 540nm excitation wavelength, a 590nm emission wavelength and a top reading protocol.

### Activated caspase 3/7 detection

Activation of Caspase 3/7 in live cultured cells was detected by cleavage of an activated caspase 3/7 recognition sequence in a DEVD four amino acid peptide conjugated to a DNA binding dye (Cellevent Caspase 3/7 ReadyProbes Reagent, Life Technologies, Melbourne). This conjugated dye is cell permeable, allowing analysis of unfixed cells, and fluoresces in the presence of activated caspase 3/7. Cells were grown in serum supplemented media before washing and addition of defined media and further growth to the point of 100 % confluence in black walled 384 well plates prior to treatments (Corning, Sigma, Sydney, Australia). Experimental treatments were applied and 1.3 drops of reagent per mL of culture media were added to the wells for the last 30 min of the experimental incubation at 37 °C in the case of chemical insults, and after light exposure in the light treated cells. No washes were applied, and uncovered well fluorescence was measured with an automatic microplate reader (Tecan F200) with a 485nm excitation wavelength, a 535nm emission wavelength and a top reading protocol.

### Annexin V and propidium iodide as markers of apoptosis and cell death

Annexin V and Propidium Iodide are widely used as markers of apoptosis and cell death respectively. They are often used together because markers of apoptosis alone may not completely reflect cellular health as necrotic cells are not identified, and unlike caspase 3 activity, Annexin V binding remains positive through a majority of the apoptotic process. In early apoptosis, phosphatidylserine is translocated to the outer leaflet of the cell plasma membrane [17], allowing annexin V labelled fluorophores to bind to apoptotic cells. In these experiments, cultured cells were prepared as described in 6 well plates and exposed to experimental treatments. Following treatments, trypsin 0.25 % (Life Technologies, Melbourne) was added to the defined treatment media to achieve cell dissociation and to preserve floating cells. The single cell suspension obtained was treated with an equal volume of 1x defined trypsin inhibitor (Life Technologies, Melbourne), centrifuged at 180 xg for 5 min at 4 °C, washed in phosphate buffered saline and resuspended to obtain 1 x 10^6^ cells/mL in 100 μL of 10mM Hepes, 140mM NaCl, 2.5mM CaCL_2_, pH 7.4. 5 μL of FITC labelled annexin V in the same solution stabilised with 0.1 % BSA and 1mM EDTA, together with 1 μL of 100 μg/L propidium iodide dead cell stain were added to each 100 μL sample. Cells were incubated for 15 min at 37 °C prior to being diluted 1:4 in the same buffer solution for analysis by flow cytometry.

### Flow cytometry

Flow cytometry was performed on a BD FACS Canto II (BD Biosciences, New Jersey). The system was calibrated prior to each experiment and 10,000 events analysed for each sample with FACS Diva Software (BD Biosciences) using control based setting of thresholds and single cell counting of fluorescent emissions at 530nm for FITC labelled Annexin V binding and greater than 575nm for propidium iodide staining. 10,000 cells were analysed for each sample of each replication. Cells positive for only annexin V were classed as early apoptotic, for both annexin V and propidium iodide as late apoptotic and those only positive for propidium iodide as necrotic.

### Statistical analysis

Data are presented as mean ± standard error of the mean. Control cells in phototoxic assays have been untreated with 9-cis retinal and shielded from light, and control cells for H_2_O_2_ and staurosporine treatments were untreated cells. Assay readings and analysis of replications in independent cultures were compared using students unpaired t-tests or repeated measures analysis of variance as appropriate. *P*-values of less than 0.05 were considered significant.

## Results

**Fig. 1:**
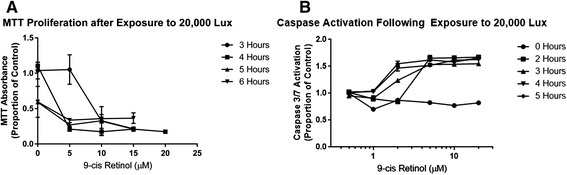
Dose and duration response of 661W photoreceptor-like cells to phototoxic stimuli in 5mM glucose. Both the MTT assay and measurement of activation of caspase 3-7 showed phototoxic stimuli had negative effects on 661W cells. These effects increased with both the duration of light exposure and concentration of the chromophore 9-cis retinal respectively. **a** MTT dye reduction shows a significant decrease in cellular reducing equivalents with increasing 9-cis retinal concentration and period of treatment in 661W cells. Phototoxic stimuli in the absence of chromophore caused a trend towards reduction in cellular reducing equivalents in 661W cells after 5-6 h, but the insult induced statistically significant differences after 4 h with the addition of 5 μM 9-cis retinal and 3 h with the addition 10 μM 9-cis retinal. **b** Caspase 3/7 activation after exposure to phototoxic stimuli also increased significantly with duration of exposure and concentration of chromophore. Caspase 3/7 activation appeared more sensitive than MTT in detecting statistically significant damage, showing negative effects after 3 h of exposure in the presence of 2 μM of the chromophore

**Fig. 2:**
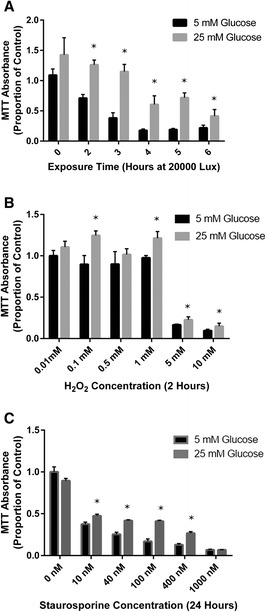
Cellular reducing equivalents are increased in cells exposed to “diabetic” glucose levels and a range of cellular insults. **a** Statistically significant increases in cellular reducing equivalents are seen in the presence of 25mM glucose after exposure to intense light at all time points between 2 and 6 h when compared to a 5mM environment. The greatest change occurred at 5 h, with 19.0 % ± 2.0 % of control reduced formazan dye levels in 5mM glucose and 72.0 % ± 8.0 % in 25mM glucose (*P* < 0.001). **b** Exposure of cells to oxidative stress in the form of H_2_O_2_ for 2 h showed a dose dependent decrease in cellular reducing equivalents in both glucose levels, however excess glucose was associated with higher levels of cellular reducing equivalents in 661W cells at all concentrations of H_2_O_2_ between 1 and 10mM. After exposure to 5mM H_2_O_2_, reduced dye density was 16.5 % ± 0.1 % of untreated controls in “non-diabetic” conditions, and 22.7 % ± 3 % in “diabetic” conditions (*P* < 0.01). **c** Staurosporine, an inducer of apoptosis, also showed a dose dependent decrease in cellular reducing equivalents in both glucose environments, but a 25mM glucose environment was associated with greater cellular reducing equivalents at all concentrations of staurosporine between 10 and 400nM. When exposed to 100nM staurosporine, reduced dye density in cells exposed to 5mM glucose was 17.0 % ± 3 % of controls, whereas in 25mM glucose reduced dye density was 27 % ± 2 % of controls (*P* < 0.01). Unstressed cells did not show any significant difference in reduced formazan dye production when exposed to different levels of environmental glucose in any of the experiments. * indicate *P* < 0.05 between cells treated in the presence of 5mM and25 mM glucose

**Fig 3:**
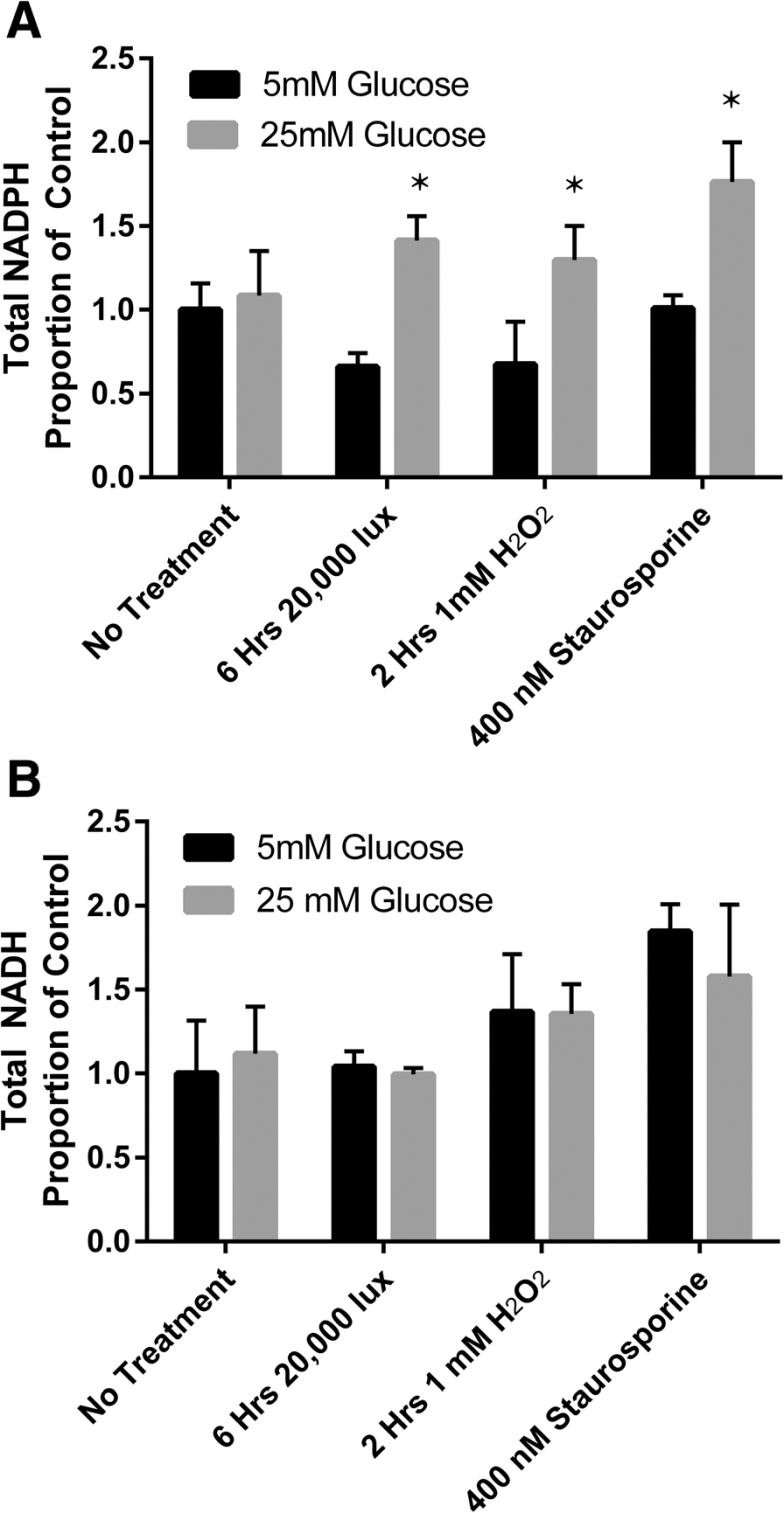
**a** Total intracellular NADPH levels in unstressed 661W photoreceptor-like cells were not significantly altered by exposure to 25mM glucose. Phototoxic stimuli and oxidative stress induced by H_2_O_2_ but not exposure to the apoptosis inducing agent reduced total intracellular NADPH levels significantly, however exposure to excess glucose significantly increased total NADPH levels in all injury models relative to similarly treated photoreceptor-like cells exposed to 5mM glucose. **b** Total intracellular NADH levels were not significantly altered by excess glucose in either untreated or injured 661W cells. * indicate *P* < 0.05 between cells treated in the presence of 5mM and 25mM glucose

661W cells were stressed with phototoxic, oxidative and apoptosis inducing insults by exposure to light in the presence of 9-cis retinal, H_2_O_2_ and staurosporine respectively. Models were validated and optimised in the presence of 5mM glucose with response curves for exposure period and dose of insult, with each insult showing a significant dose-response relationship as assessed by both the MTT assay and caspase 3/7 activity. Threshold points for maximum change were noted after 4 h of exposure to 20,000 lux and 2 μM 9-cis retinal (Fig. 1), and confirmed widely reported testing thresholds of 2 h of exposure to 1mM H_2_O_2_ and 24 h of exposure to 100-400nM Staurosporine (Figs. 1, 2 and 3).

### Cellular reducing equivalents

The MTT proliferation assay was used to assess the cellular reducing power of stressed photoreceptor-like cells in the presence of different levels of glucose as an energy substrate. In the case of each insult, 25mM glucose was found to statistically significantly increase the cellular reducing equivalents of the neurons over a range of insult doses. Exposure to 25mM glucose was associated with no statistically significant change in total cellular reducing equivalents, as measured by MTT reduction, for up to 3 h of exposure to light, whereas cells exposed to 5mM glucose showed statistically significant reductions in cellular reducing potential after 2 h. At each time point from 2 to 6 h, the MTT assay showed a statistically significant positive effect of “diabetic” levels of glucose on cellular reducing potential when compared to non-diabetic glucose conditions (Fig. 2a).

Similarly, after exposure to oxidative insult in the form of 2 h of H_2_O_2_, the availability of 25mM glucose as an energy substrate was associated with a statistically significant positive effect on 661W photoreceptor reducing capacity as measured by the MTT assay over a range of insult concentrations. Statistically significant increases in the MTT measure of cellular reducing equivalents was seen with excess glucose treatment over all tested concentrations of H_2_O_2_ from 1mM to 10mM, with no significant difference noted at lower concentrations, where the insult itself produced little effect (Fig. 2b).

The same pattern was seen when 661W photoreceptor-like cells were treated with the apoptosis inducing agent staurosporine. In this case, higher glucose substrate levels were associated with statistically significant increases in reducing potential of cells subjected to similar staurosporine insults. This was noted after 24 h of exposure to all tested staurosporine concentrations ranging from 10 to 400nM (Fig. 2c). When treated with 1000nM of staurosporine, cellular death rate was high and no significant difference between the tested environmental glucose levels could be measured.

### Reduced NADP(H) and NAD(H) levels

NADH and NADPH are two coenzymes which are major contributors to cellular reducing potential as measured by the MTT method. Exposure to 25mM glucose did not significantly alter NADPH levels measured in untreated 661W photoreceptor-like cells. Cells subjected to each injury model showed significantly reduced cellular NADPH levels in the presence of 5mM glucose, however exposure to 25mM glucose significantly increased cellular NADPH in each case (Fig. 3a). Exposure to 25mM glucose did not significantly alter cellular levels of NADH in untreated cells or in any of the models of injury (Fig. 3b).

### Caspase 3/7 activation

**Fig. 4:**
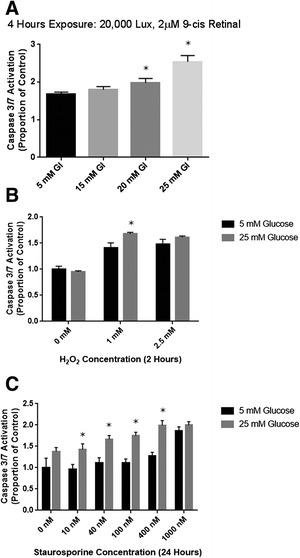
Caspase 3/7 activity in 661W photoreceptor-like cells after various cellular insults. **a** Increasing concentrations of environmental glucose were associated with a trend of increasing caspase 3/7 activation in response to phototoxic stimuli which became significant in 25mM glucose. Caspase 3/7 cleavage of the fluorescently labelled DEVD peptide was increased 168 %  ± 5 % in 5mM glucose relative to untreated controls, but “diabetic” conditions was associated with a 253 % ± 17 % increase in caspase 3/7 activation (*P* < 0.01). **b** Oxidative stress in the form of H_2_O_2_ increased caspase 3/7 activation in both 5mM and 25mM glucose. 2 h exposure to 1mM H_2_O_2_ was associated with a 140 % ± 10 % increase in caspase 3/7 activation relative to control in a low glucose environment, and this increased to 168 ± 3 % in the higher glucose environment (*P* < 0.05). 2.5mM H_2_O_2_ treatment was not associated with a significant change in activated caspase 3/7 in the different glucose environments at this time point (*P* < 0.16). **c** Staurosporine, an inducer of apoptosis, caused a dose dependent increase in caspase 3/7 activation at both low and high glucose levels. However, caspase activation was significantly greater in the 25mM glucose environment at staurosporine concentrations between 10 and 400nM. When treated with 100nM staurosporine, 661W cells showed a 127 % ± 9 % increase in caspase 3/7 activation in “non-diabetic” conditions, and this increased to 199 % ± 8 % in diabetic conditions (*P* < 0.01). Untreated cells did not show any significant difference in caspase 3/7 activation when exposed to different levels of environmental glucose in any of the experiments. Incubations for other periods confirmed that these results represented a true acceleration of caspase activation in the cultures exposed to excess glucose (not shown). * indicate *P* < 0.05 between cells treated in the presence of 5mM and 25mM glucose

Conversely, fluorescent detection of activated caspase 3 and caspase 7 was associated with smaller differences between stressed cells treated in the presence of high or low glucose. However caspase activity was greater in the presence of the same range of insults when glucose levels were higher, indicating a negative effect of glucose on the neurons by this measure. Exposure to 25mM glucose was associated with increased activation of caspase 3/7 when cells were exposed to 20,000 lux white light (Fig. 4a). Similarly, oxidative insult in the form of 2 h exposure to H_2_O_2_ was associated with a statistically significant increases in activation of caspase 3/7 in the presence of excess glucose (Fig. 4b). Cell stress in the form of 24 h treatment with the apoptosis inducing agent staurosporine also showed significantly increased activation of caspase 3/7 in the presence of excess glucose, with statistically significant negative effects associated with higher glucose levels noted at all concentrations between 10 and 400nM (Fig. 4c). Once again, at 1000nM, high levels of caspase 3/7 activation in both high and low glucose conditions did not allow any measurable differences to be detected.

### Annexin V and propidium iodide flow cytometry

**Fig. 5:**
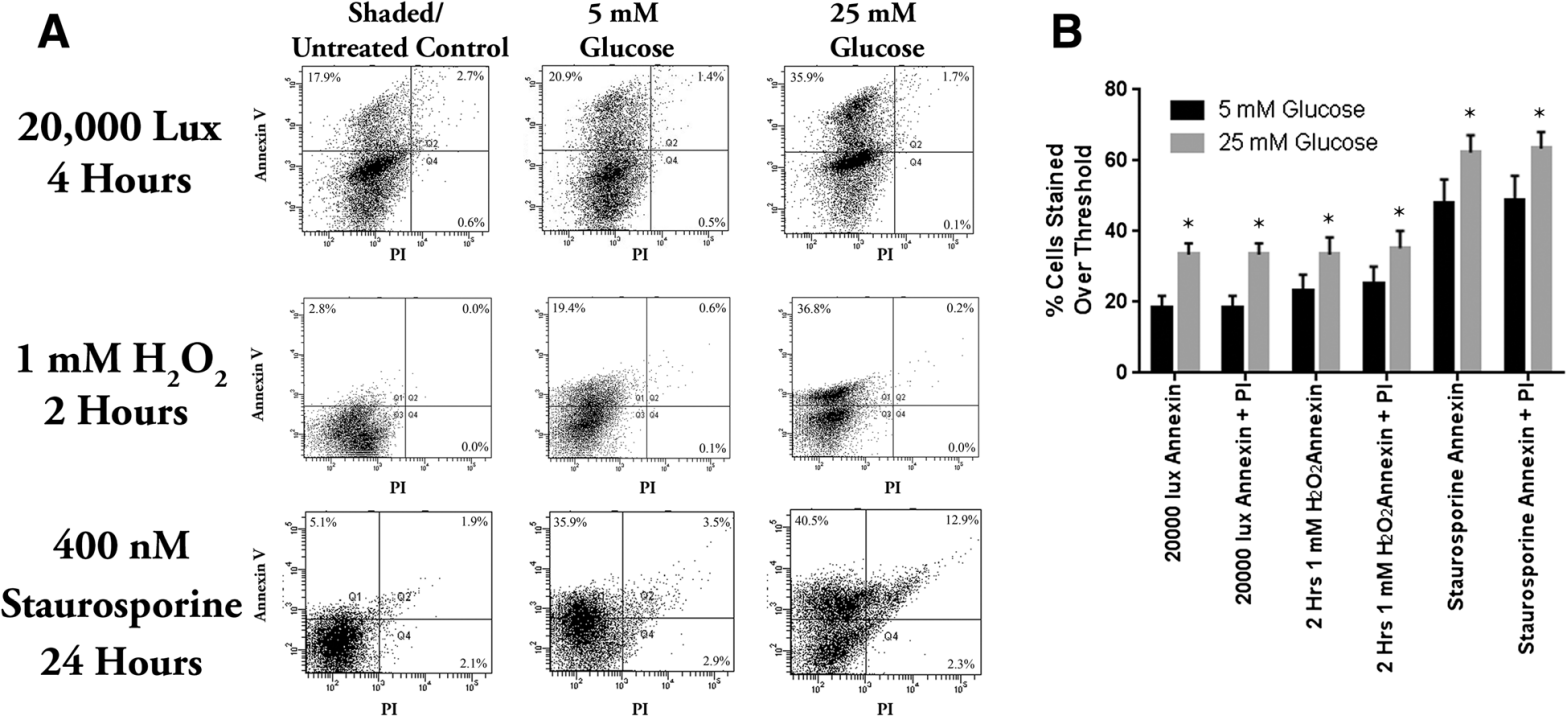
Flow cytometry characterisation of cell death by apoptosis (Annexin V binding) and necrosis (Propidium Iodide marking). **a** Representative flow cytometry output. Stained untreated or shaded controls showed significantly less binding of Annexin V or marking with PI relative to treated cells for all insults and conditions. None of the insults induced a significant degree of primary necrosis, with some PI marking of late apoptotic cells particularly in cells treated with staurosporine for 24 h. A clear second population of Annexin V labelled apoptotic cells was evident in stressed cells, with the separation between the apoptotic and non-apoptotic populations becoming more pronounced in the higher glucose environment. **b** A high glucose environment was associated with significantly higher levels of binding for Annexin V (early apoptotic cells) and combined Annexin V/PI (ie total of apoptotic, necrotic and late apoptotic/necrotic cells) than low glucose conditions for all insults. * indicate *P* < 0.05 between cells treated in the presence of 5mM and 25mM glucose

To confirm these results of increasing cell damage in the presence of high glucose levels, flow cytometry characterisation of cell death by apoptosis (Annexin V binding) and necrosis (Propidium Iodide marking) was performed on replications of exactly 10,000 cells for each of the three different insults. Results with representative outputs are shown in Fig. 5. All insults used in this study showed little ability to induce necrosis, with most negatively affected cells displaying characteristics of either early or late apoptosis (Fig. 5a). For the three different tested cellular insults (phototoxicity, oxidative stress via H_2_O_2_ and apoptosis induction with staurosporine), 661W photoreceptor-like cells were statistically significantly less resilient in the presence of diabetic levels of glucose (25mM) when compared to non-diabetic levels (5mM) (Fig. 5b).

## Discussion

Identifying useful in vitro models of the effect of stress on neurons in diabetic conditions is important if the mechanisms of diabetic retinal neuropathy or other complications of diabetes are to be explored and perhaps eventually targeted therapeutically. The results presented here show that glucose reduces the resilience of 661W photoreceptor-like cells to a range of insults when cellular health is measured by caspase activation, Annexin V expression and propridium iodide expression, all methods which do not use cellular energy production as a surrogate for cell survival.

These results also show that cellular reducing equivalents are increased in stressed 661W cells by higher glucose substrate concentrations, but only in conditions of stress. By increasing cellular energy production or cellular reducing equivalents either by providing an over-abundance of input into cellular energy pathways or altering the balance of pathways a particular cell type uses for energy production (for example increasing the flux through the pentose phosphate shunt) [8, 28], glucose availability could well act in unpredictable ways on total cellular reducing equivalents. Early reports have shown that cellular reducing equivalents as measured by MTT reduction did in fact depend on glucose concentration in seven different tumour cell lines, with those most dependent upon glucose for energy showing the greatest sensitivity [29]. The results presented here did not show a similar significant change in MTT reduction in 661W cells when exposed to different levels of glucose in an unstressed state, but the phenomenon became evident when the cells were stressed by three different mechanisms. Nonetheless, the increase in total cellular reducing equivalents in stressed retinal neurons is not associated with neuroprotection, is decoupled from cellular health markers, and is associated with greater levels of cell death in these models.

The mechanism of this increase in cellular reducing equivalents induced by the availability of high glucose levels in these models appears to partly be an increase in NADPH but not NADH levels, which remained stable. This indicates that increased NADPH is likely to be partially responsible for the decoupling of cellular reducing power and cellular survival caused by increased glucose availability in these models of injury. These findings are supported by previous studies which have shown that NADPH levels are also increased in mixed primary retinal cell cultures exposed to high levels of glucose [8], partly due to diversion of energy substrates to the pentose phosphate shunt, the cell’s major source of NADPH.

The decoupling of cellular reducing potential and cellular survival associated with exposure to “diabetic” levels of glucose has important implications in both laboratory science and in translational efforts to treat diabetic retinal neuropathy. In the laboratory it shows that proliferation assays may not be an accurate measurement of cell health when used in investigations designed to explore the effect of hyperglycaemia on cellular survival in the retina, or indeed in other parts of the body. In translational research, if this decoupling phenomenon is also found in the retina in vivo, it could indicate that targeting diabetic retinal neuropathy by enhancing cellular reducing ability or with antioxidant agents may not necessarily improve retinal neuronal survival in diabetic retinal neuropathy.

The difficulty in interpreting proliferation assays when measured in the presence of different levels of energy substrates is expected when the mechanism of the MTT reduction assay and its modifications are considered. The assay is based on the dye binding studies of Slater et al. [30], in which tetrazolium salts formed colorimetric formazan endproducts by accepting electrons from cellular reducing equivalents (NADH, NADPH and others). This is thought to occur in part via the action of mitochondrial dehydrogenases [30], which exists in the mitochondrial respiratory chain. It is for this reason that the MTT measure is considered a proxy measure of cellular energy production, and since the biochemistry of energy production cannot occur in dead cells, the assay has been shown to be an accurate estimate of cellular viability in the presence of stable levels of energy substrates [15]. Such an interpretation in this investigation would have erroneously indicated that glucose has a protective effect in these three models of photoreceptor injury.

These investigations were performed on 661W photoreceptor-like cells and it is likely that other cells with different energy substrate requirements may not exhibit this decoupling of cellular reducing potential and cellular survival, or may not display it to the extent shown in these cells. This includes other cells of retinal origin, particularly retinal glial cells but also other retinal neurons or non-transformed photoreceptors. A range of different insults was used in in these investigations, but it is additionally possible that other types of injuries to 661W cells exposed to high glucose levels may not exhibit the phenomenon of increase cellular reducing potential in the absence of neuroprotection seen in this manuscript. Nonetheless these results emphasise the importance of supporting data from the use of proliferation assays in diabetic investigations with non-energy dependent measurements of cellular health or survival.

## Conclusion

The presented results indicate that increased production of cellular reducing equivalents, in particular NADPH, in stressed photoreceptor-like cells exposed to physiologically high levels of glucose is associated with reduced cellular resilience against a variety of insults. Therefore, the increased intracellular reducing potential induced by high glucose levels in retinal cells may be overcome by other, non-redox associated deleterious effects of excess glucose. These results indicate that the use of energy dependent surrogates of cellular health need to be carefully considered if energy substrate levels are to be altered in experimental protocols, and that translational efforts directed towards preventing diabetic retinal neuropathy by reducing oxidative stress may not necessarily result in neuroprotection.
